# Hypothalamic Orexin-A Neurons Are Involved in the Response of the Brain Stress System to Morphine Withdrawal

**DOI:** 10.1371/journal.pone.0036871

**Published:** 2012-05-09

**Authors:** M. Luisa Laorden, Szilamér Ferenczi, Bernadett Pintér-Kübler, Laura L. González-Martín, M. Carmen Lasheras, Krisztina J. Kovács, M. Victoria Milanés, Cristina Núñez

**Affiliations:** 1 Cellular and Molecular Pharmacology Laboratory, Faculty of Medicine, Murcia, Spain; 2 Instituto Murciano de Investigación Biosanitaria (IMIB), Murcia, Spain; 3 Molecular Neuroendocrinology Laboratory, Institute of Experimental Medicine, Budapest, Hungary; Institut National de la Santé et de la Recherche Médicale, France

## Abstract

Both the hypothalamus-pituitary-adrenal (HPA) axis and the extrahypothalamic brain stress system are key elements of the neural circuitry that regulates the negative states during abstinence from chronic drug exposure. Orexins have recently been hypothesized to modulate the extended amygdala and to contribute to the negative emotional state associated with dependence. This study examined the impact of chronic morphine and withdrawal on the lateral hypothalamic (LH) orexin A (OXA) gene expression and activity as well as OXA involvement in the brain stress response to morphine abstinence. Male Wistar rats received chronic morphine followed by naloxone to precipitate withdrawal. The selective OX1R antagonist SB334867 was used to examine whether orexins' activity is related to somatic symptoms of opiate withdrawal and alterations in HPA axis and extended amygdala in rats dependent on morphine. OXA mRNA was induced in the hypothalamus during morphine withdrawal, which was accompanied by activation of OXA neurons in the LH. Importantly, SB334867 attenuated the somatic symptoms of withdrawal, and reduced morphine withdrawal-induced c-Fos expression in the nucleus accumbens (NAc) shell, bed nucleus of stria terminalis, central amygdala and hypothalamic paraventricular nucleus, but did not modify the HPA axis activity. These results highlight a critical role of OXA signalling, via OX1R, in activation of brain stress system to morphine withdrawal and suggest that all orexinergic subpopulations in the lateral hypothalamic area contribute in this response.

## Introduction

Orexin-A (OXA) and Orexin-B (OXB) (also known as hypocretin-1 and hypocretin-2, respectively) are neuropeptides synthesized exclusively in hypothalamus that are involved in several physiological processes [1], [2]. Two G protein-coupled receptors have been identified for the orexin system, orexin receptor type 1 (OX1R) and orexin receptor type 2 (OX2R). Whereas OX1R binds OXA with higher affinity (30 nM) than OXB, OX2R binds both orexins with equal affinity [1]. Generally, it is thought that OX1R is coupled to G_q_, and OX2R is coupled to G_q_ or G_i_/G_o_, but signalling mechanisms are cell specific and has not been systematically studied. Orexinergic neurons are localized in the lateral hypothalamus (LH): the lateral region (LLH), perifornical area (PFA), and dorsomedial hypothalamus (DMH) [1], [3], [4], while their projections are widely distributed in many brain areas [4]. Orexinergic neurons arising in the DMH and PFA project to brainstem nuclei and involved in control of arousal and modulation of stress responses [5]. Neurons from the LLH project to brain areas broadly related to positive reinforcing effects of drugs (ventral tegmental area, VTA, nucleus accumbens, NAc; [4], [5]). So, orexins have been lately related to reward and addiction processes.

Several neurotransmitters/neuromodulators, including corticotropin- releasing factor (CRF), ghrelin, neurotensin, vasopressin and oxytocin excite OXA neurons. On the other hand, noradrenaline (NA), dopamine (DA), serotonin, neuropeptide Y, and leptin inhibit orexinergic cells [6]. CRF is widely distributed throughout the brain and plays a major role in coordinating the behavioral and autonomic responses to stress [7]. CRF is released from two brain regions to participate in two separate (but connected) stress systems. In the hypothalamo-pituitary-adrenal (HPA) axis stress system, CRF neurons in the parvocellular region of the hypothalamic paraventricular nucleus (PVN) regulate the release of pituitary adrenocorticotropic hormone (ACTH) and adrenal glucocorticoids. In the extrahypothalamic stress system, CRF neurons in the central nuleus of amygdala (CeA) and in the bed nucleus of the stria terminalis (BNST) excite noradrenergic neurons in the locus coeruleus (LC) and the nucleus of the solitary tract (NTS). Increased activity of this ascending noradrenergic system results in activation of CeA, BNST and PVN neurons. The activation of the CRF neurons of the PVN is also associated with increased activity in the NTS [8], [9]. In addition, CRF has been reported to contribute to the anxiogenic and adverse symptoms of withdrawal from exposure to several drugs of abuse, including cocaine and opiates [9], [10].

Recent investigations revealed participation of the orexinergic system in brain stress system [11]. The present study was designed to evaluate changes in the orexinergic system during naloxone-precipitated morphine withdrawal. We addressed the role of orexinergic inputs in the development of somatic signs of the opiate abstinence syndrome and the brain stress systems responses to morphine withdrawal.

It has been reported that OXA activates PVN neurons [12] and that intracerebroventricular (i.c.v.) OXA increases plasma levels of glucocorticoids and ACTH [13]. However, it remains to be determined whether OXA modulates the activity of the extrahypothalamic CRF neurons. Further aim of this work was to confirm the orexin projections to the main nuclei of the brain stress system, such as NAc shell, BNST, PVN, CeA, and NTS, and to study the effect of pharmacological blockade of the orexinergic system, by OX1R antagonist (SB334867) administration, on the somatic symptoms produced during morphine withdrawal. In addition, c-Fos expression in the brain stress system and glucocorticoids release during morphine withdrawal were measured in morphine-dependent SB334867-treated rats.

## Results

### OX1R antagonist SB334867 attenuates somatic expression of naloxone-precipitated morphine withdrawal

**Figure 1:**
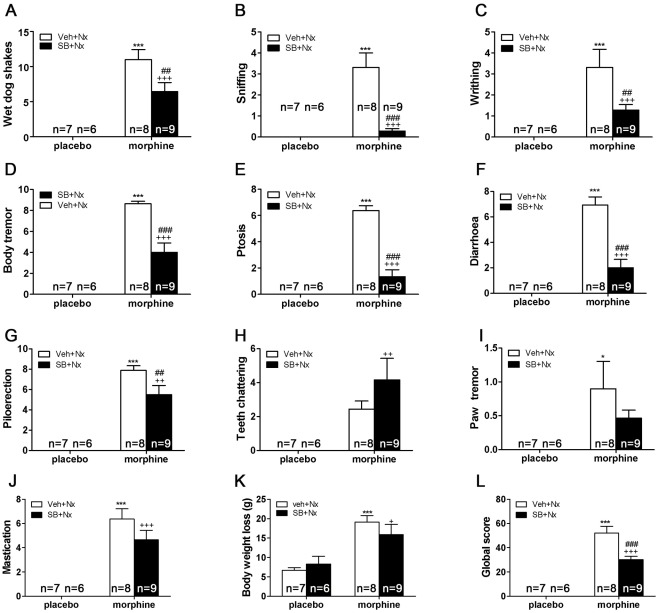
Attenuation of the severity of somatic signs of naloxone-precipitated morphine withdrawal up to 30 min after the naloxone injection by SB-334867 pretreatment. The following variables were counting: (A) wet-dog shakes; (B) sniffing; (C) writhing; (D) body tremor; (E) ptosis; (F) diarrhoea; (G) piloerection; (H) mastication; (I) teeth chattering; (J) paw tremor; (K) body weight loss. Somatic signs of withdrawal were observed during 30 min immediately after naloxone injection (1 mg/kg s.c.) A global withdrawal score (L) was calculated for each animal as described in Methods. Data are expressed as mean ± SEM. *p<0.05; ***p<0.001, versus placebo + vehicle (veh) + naloxone (nx); ^++^p<0.01; ^+++^p<0.001 versus similar groups receiving vehicle instead of SB-334867.

Six days after the implantation of morphine or placebo pellets, rats were challenged with naloxone (1 mg/kg s.c.) and immediately tested for the occurrence of somatic signs of opiate withdrawal. The following somatic signs were significantly present in morphine-treated groups (n = 8) when compared with placebo-treated groups (n = 7): wet dog shakes (p<0.001), sniffing (p<0.001), writhing (p<0.001), body tremor (p<0.001), ptosis (p<0.001), diarrhea (p<0.001), piloerection (p<0.001), teeth chattering (p<0.001), paw tremor (p<0.05), mastication (p<0.001), and body weight loss (p<0.001; n = 10). The analysis of the global withdrawal score confirmed these differences between morphine- and placebo-treated rats (p<0.001). The results for two-way ANOVA analysis are shown in Table 1. When OX1R receptors were blocked, comparisons between morphine (n = 9) groups showed that wet dog shakes (p<0.001), sniffing (p<0.001), writhing (p<0.01), body tremor (p<0.001), ptosis (p<0.001), diarrhea (p<0.001), piloerection (p<0.01), and mastication (p<0.001) were significantly decreased in morphine-dependent rats receiving SB334867 before naloxone (Table 1, Figure 1A–K). The analysis of the global withdrawal score confirmed that SB334867 significantly reduced somatic expression of withdrawal in morphine-treated rats (p<0.001; Table 1, Figure 1L). Thus, the blockade of OX1R overall decreased the expression of naloxone-precipitated somatic signs of opiate withdrawal, reducing global scores of morphine-dependent SB334867-treated rats. No changes were seen in the placebo-treated group receiving SB334867 (n = 6) and the control group injected with vehicle.

**Table 1:** SB334867 attenuates the somatic expression of naloxone-precipitated morphine withdrawal.

	*Two-Way ANOVA*				
	*Chronic treatment (morphine vs placebo)*		*Pretreatment (SB334867 vs vehicle)*		*Interaction*
*Signs*	*F _1,27_*	*p<*	*F_1,26_*	*p<*	*F_1,26_ p<*
Wet dog shakes	61.39	0.0001	4.19	n.s	4.19 n.s
Paw tremor (P25)	10.62	0.0032	1.07	n.s	1.07 n.s
Sniffing	22.32	0.0001	15.94	0.0005	15.94 0.0005
Writhing	21.35	0.0001	4.20	n.s	4.20 n.s
Tremor	122.75	0.0001	16.47	0.0004	16.47 0.0004
Ptosis	101.57	0.0001	43.45	0.0001	43.45 0.0001
Mastication	70.96	0.0001	1.70	n.s	1.70 n.s
Teeth chatering	16.08	0.0005	1.10	n.s	1.10 n.s
Piloerection	120.27	0.0001	3.79	n.s	3.79 n.s
Diarrhea	70.86	0.0001	21.63	0.0001	21.63 0.0001
Weight loss (P31)	28.42	0.0001	0.18	n.s	0.18 n.s
Global score (P27)	124.27	0.0001	8.66	0.0066	8.66 0.0066

Two-way ANOVA with chronic treatment (morphine vs. placebo) and pretreatment before naloxone (SB334867 vs. vehicle) as between-subjects factors. When significant interactions in pretreatment or between these two factors were observed, a subsequent *post hoc* test was applied.

### Naloxone-precipitated morphine withdrawal induces orexin gene expression

**Figure 2:**
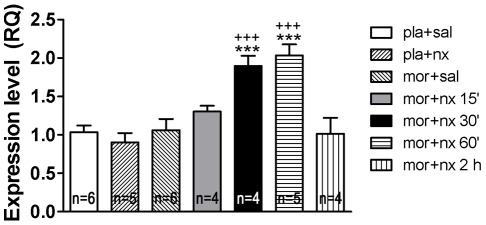
Morphine withdrawal induced OXA gene expression in the hypothalamus. Rats were sacrificed at different time-points (15, 30, 60 and 120 min) after saline or naloxone injection. Mean ± SEM RQ (relative quantification of the comparative C_T_ experiments) expression values were obtained by real-time qPCR measurement, where the expression of OXA gene in morphine withdrawn rats is expressed relative to the placebo implanted controls receiving naloxone. ***p<0.001 versus placebo + naloxone; ^+++^p<0.001 versus morphine + saline.

Next, we assessed if activation of orexinergic neurons in response to chronic morphine treatment and precipitated withdrawal results in induction of orexin gene expression. Time course of changes in OXA mRNA levels was followed in the hypothalamic samples by quantitative RT-PCR (Figure 2). When compared to placebo implanted controls (n = 6) no change in mRNA levels was detected in response to chronic morphine (n = 6) or to naloxone in morphine-naïve animals (n = 5). Two-way ANOVA showed that there was a significant morphine pretreatment main effects (30 min: F(1,17) = 16.38, p = 0.0008; 60 min: F(1,18) = 20.80, p = 0.0002), a significant naloxone main effect (30 min: F(1,17) = 7.803, p = 0.0125; 60 min: F(1,18) = 10.98, p = 0.0039) and a significant interaction “morphine pretreatment” X “SB treatment” (30 min: F(1,17) = 14.80. p = 0.0013; 60 min: F(1,18) = 19.03, p = 0.0004). Neuman Keuls' post hoc test showed that at 30 (n = 4) and 60 (n = 5) min after naloxone injection there was a significant increase mRNA expression compared with placebo groups receiving naloxone (p<0.001; p<0.001, respectively) and with morphine-dependent animals receiving saline instead naloxone (p<0.001; p<0.001, respectively).

### Chronic morphine treatment and naloxone-precipitated morphine withdrawal do not modify the number of OXA neurons in the LH

**Figure 3:**
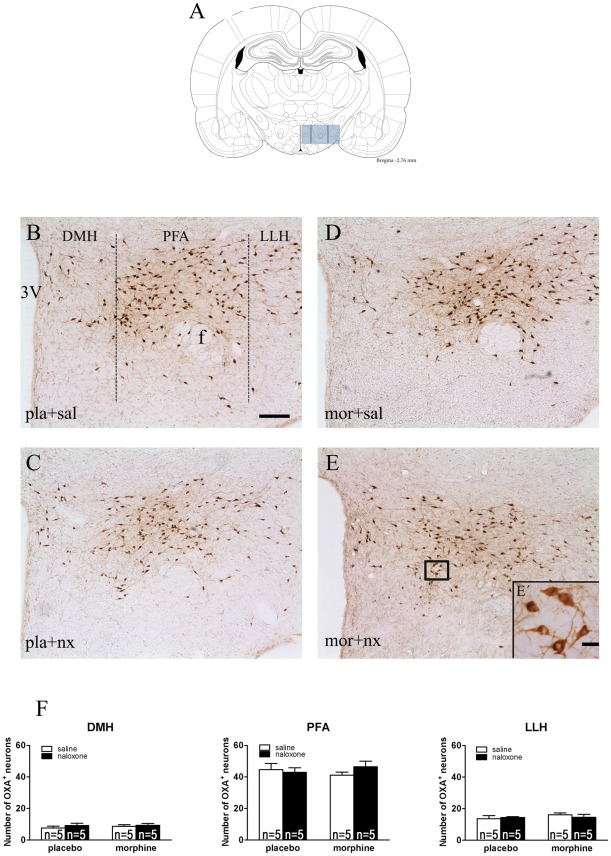
Chronic morphine and morphine withdrawal do not modify the number of OXA neurons in the DMH, PFA and LLH regions of the lateral hypothalamus. Placebo and morphine-dependent groups were sacrificed 120 min after saline or naloxone administration. (A) Schematic anatomic representation of LH subdivisions adapted from Paxinos and Watson stereotaxic atlas [42]. (B–E, E′) Representative photographs illustrating regional orexin A cell expression in the LH. (F) Mean ± SEM in the three regions of the LH. 3V, third ventricle; f, fornix. Scale bars, 200 μm (B–E) and 20 μm (E′).

To evaluate if chronic morphine and naloxone-precipitated morphine withdrawal differentially affects the number of OXA neurons in the LH, the cell counts of OXA immunoreactive profiles were taken in three sub-population of the LH: DMH, PFA and LLH. The two-way ANOVA performed on the number of OXA-positive neurons in placebo (n = 5)- and chronic morphine-treated rats (n = 5), with or without naloxone injection, indicated that there was not a significant morphine pretreatment main effects, no significant naloxone main effect nor a significant interaction [DMH: morphine pretreatment, F(1,16) = 0.25; p = 0.6271; naloxone injection, F(1,16) = 0.91; p = 0.3556; interaction, F(1,16) = 0.14; p = 0.7143. PFA: morphine pretreatment, F(1,16) = 0.00; p = 0.9863; naloxone injection, F(1,16) = 0.32; p = 0.5979; interaction, F(1,16) = 1.28; p = 0.2754. LLH: morphine pretreatment, F(1,16) =  0.90; p = 0.3569; naloxone injection, F(1,16) =  0.06; p = 0.8174; interaction, F(1,16) = 0.67; p = 0.4246]. As depicts in Figure 3F, chronic morphine or naloxone injection did not significantly alter the number of OXA neurons in the LH.

### Naloxone-precipitated morphine withdrawal activates OXA neurons

**Figure 4:**
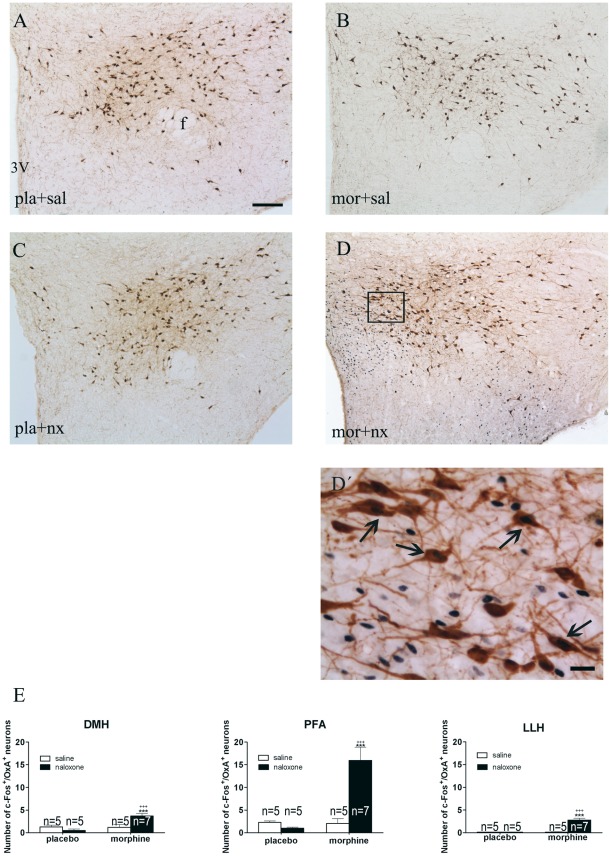
Naloxone-induced morphine withdrawal results in activation of orexin A cells in the DMH, PFA and in the LLH. (A–D) Representative photographs showing double-label immunohistochemistry for c-Fos and OXA in the LH. (D′) High magnification image showing c-Fos-positive (black)/OXA-positive (brown) neurons. 3V, third ventricle; f, fornix. Scale bars, 200 μm (A–D) and 50 μm (D′). Arrows indicate c-Fos in OXA-positive neurons. (E) Regional expression of c-Fos in OXA positive cells. Bars represent the mean ± SEM. ^+++^p<0.001 versus morphine + saline; ^***^p<0.001 versus placebo + naloxone.

c-Fos protein, the product of c-fos immediate early gene, has been used as a marker for neuronal activation. To reveal if OXA neurons are activated during naloxone-precipitated morphine withdrawal, c-Fos and OXA immunostaining was co-localized and quantified in sections of the lateral hypothalamic area (Figure 4A–D). Two-way ANOVA revealed main effect of morphine treatment [DMH: F(1,18) = 8.98; p* = *0.0077; PFA: F(1,18) = 13.74; p = 0.0016; LLH: F(1,18) = 28.20; p<0.0001], naloxone injection [DMH: n.s.; PFA: F(1,18) = 10.04; p = 0.0053; LLH: F(1,18) = 26.82; p<0.0001], and interaction between pretreatment and acute treatment [DMH: F(1,18) = 9.79; p* = *0.0058; PFA: F(1,18) = 14.70; p = 0.0012; LLH: F(1,18) = 27.10; p<0.0001]. *Post hoc* analysis revealed that morphine withdrawal (n = 7) increased (p<0.001) the number of OXA-containing neurons expressing c-Fos in all three subpopulation of the LH compared with placebo controls receiving naloxone (n = 5) and morphine-treated rats injected with saline (n = 5) instead of naloxone (Figure 4E). Administration of the opioid antagonist to control rats did not induce any modification, compared with control groups receiving saline (n = 5).

### OX1R antagonism attenuates naloxone-precipitated morphine withdrawal-evoked activation of extrahypothalamic and hypothalamic brain stress systems

**Figure 5:**
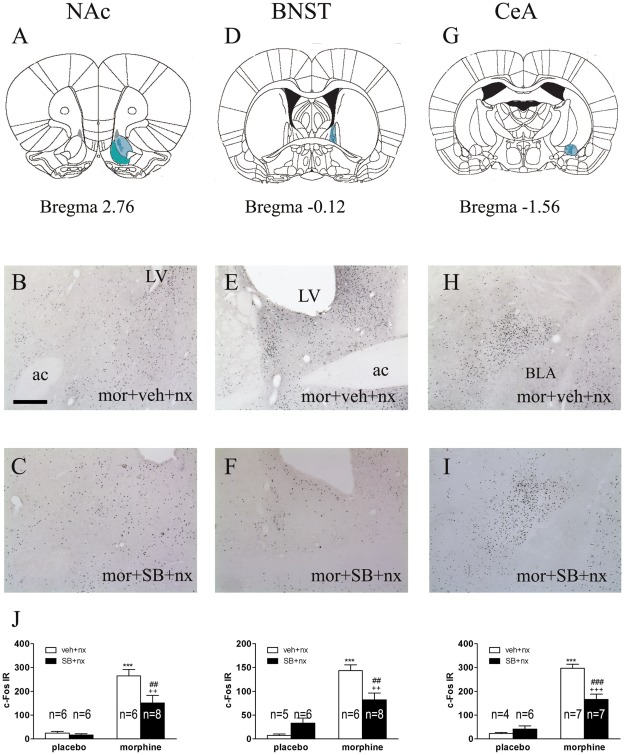
Naloxone-induced morphine withdrawal results in activation of extended amygdala. SB-334867 attenuated c-Fos expression in the NAc, BNST and CeA in morphine-withdrawn rats. Rats were pretreated with the selective OX1R antagonist SB334867 20 min prior naloxone injection and were sacrificaed 120 min after naloxone administration. (A, D, G) Schematic anatomic representation of NAc shell, BNST and CeA adapted from Paxinos and Watson atlas (2007). Labeled areas delineate regions where c-Fos expression was examined. (B I) Representative photographs of c-Fos expression in the NAc, BNST (oval) and CeA in animals pretreated with morphine-vehicle-naloxone (B, E, H) or with morphine-SB-334867-naloxone (C, F, I). LV, lateral ventricle; ac, anterior comissure; BLA, basolateral amygdala. Scale bar, 200 µm. (J) Quantification of neurons expressing c-Fos in the NAc shell, BNST (oval) and CeA. Bars represent mean ± SEM. ***p<0.001 versus placebo + vehicle (veh) + naloxone (nx); ^++^p<0.01; ^+++^p<0.001 versus placebo + SB-334867+ naloxone; ^##^p<0.01, ^###^p<0.001 versus morphine + vehicle + naloxone.

**Figure 6:**
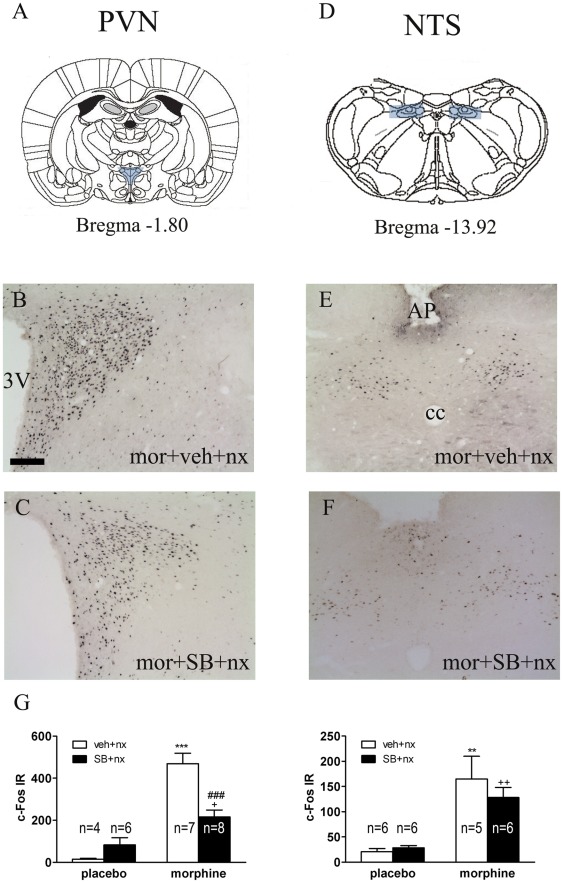
SB334867 attenuated naloxone-induced morphine withdrawal induction of c-Fos expression in the PVN but not in the NTS. (A, D) Schematic anatomic representation of the PVN and NTS adapted from Paxinos and Watson atlas (2007). Labelled areas delineate regions where c-Fos expression was examined. (B–F) Representative photographs of c-Fos expression in the PVN and NTS in animals pretreated with morphine-vehicle-naloxone (B, E) or with morphine-SB334867-naloxone (C, F). Animals were sacrificed 120 min after naloxone administration. 3V, third ventricle; AP, area postrema. Scale bar: 100 µm. (G) Quantification of neurons expressing c-Fos in the PVN (parvocellular subdivision) and in the NTS-A_2_ catecholaminergic cell group. Bars represent mean ± SEM. **p<0.01 ***p<0.001 versus placebo + vehicle (veh) + naloxone (nx); ^+^p<0.05; ^++^p<0.01 versus placebo + SB-334867+ naloxone; ^###^p<0.001 versus morphine + vehicle + naloxone.

We assessed the influence of the OX1R antagonist SB334867 on c-Fos expression in the brain stress system (NAc shell, BNST and CeA; Figure 5) as well as on the PVN and NTS-A_2_ noradrenergic cell group (Figure 6). Two-way ANOVA indicated that there were significant effects of morphine pretreatment, SB334867 administration and “morphine pretreatment” X “SB treatment” interaction on c-Fos expression in the NAc shell [morphine pretreatment: F(1,22) = 59.45; p<0.0001; SB treatment: F(1,22) = 6.35; p = 0.0195; interaction: F(1,22) = 4.77; p = 0.0400], CeA [morphine pretreatment: F(1,20) = 114.76; p<0.0001; SB treatment: F(1,20) = 9.17; p = 0.0066; interaction: F(1,20) = 16.26; p = 0.0007], and PVN [morphine pretreatment: F(1,21) = 53.84; p<0.0001; SB treatment: F(1,21) = 5.35; p = 0.0310; interaction: F(1,21) = 16.11; p = 0.0006]. The two-way ANOVA also indicated that there were significant effect of morphine pretreatment and “pretreatment” X “SB treatment” interaction on c-Fos expression in the BNST [pretreatment: F(1,21) = 55.90; p<0.0001; Interaction: F(1,21) = 12.41; p = 0.0020] and significant effect of morphine pretreatment in the NTS [F(1,19) = 29.83; p<0.0001].

*Post hoc* analysis (Figure 5J) revealed that morphine withdrawal increased (p<0.001) c-Fos expression in the extended amygdala: NAc shell; (Figure 5B; n = 6), BNST (Figure 5E; n = 6) and CeA (Figure 5H; n = 7). We observed similar significant increase in c-Fos expression in the PVN (p<0.001; n = 7) and NTS (p<0.01; n = 5). We also observed that the main effect of SB334867 administration was to decrease c-Fos expression. This effect was significant across extended amygdala areas (Figure 5C, F, I, J; NAc, n = 8; BNST, n = 8) and the PVN (Figure 6B, C; n = 8).

### OXA fibers project to the extended amygdala, PVN and NTS

**Figure 7:**
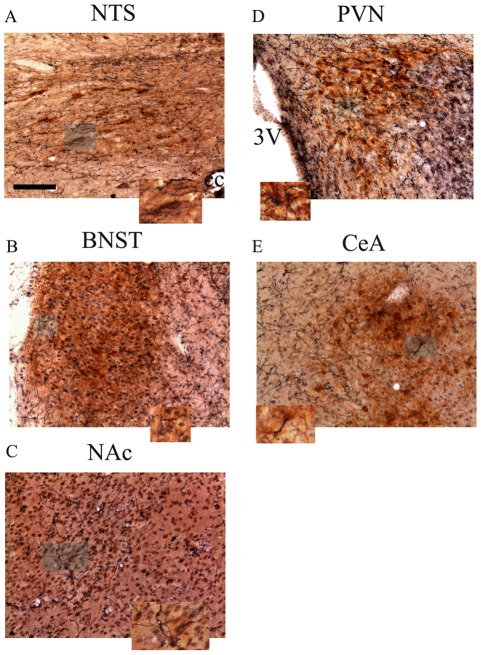
Immunohistochemical localization of orexin A nerve fibers in the NTS, BNST, NAc, PVN and CeA in placebo-implanted control rats. Representative photographs showing double-label immunohistochemistry for TH and OXA (NTS, A), CRF and OXA (BNST, B; PVN, D; and CeA, E) and pro-DYN and OXA (NAc, C). Scale bar, 50 µm. Images depict the orexin A innervations (blue-black) of the CRF, pro-DYN and TH neurons (brown) in the extended amygdala, NTS and PVN. Higher magnification images of the boxes areas depicted in each photomicrograph show examples of the areas where OXA axons are opposed to the somata of CRF, TH and pro-DYN cells. Abbreviations: NTS, nucleus of the solitary tract; BNST, bed nucleus of the stria terminalis; NAc, nucleus accumbens; PVN, hypothalamic paraventricular nucleus; CeA, central amygdala; cc, canal central; 3V, third ventricle.

Whereas OXA cell bodies are restricted to the LH, OXA nerve fibers project widely into the extended amygdala (NAc shell, BNST and CeA), PVN, and NTS, areas that are critically involved in addiction and brain stress system (Figure 7). A double-label immunohistochemical staining was carried out to investigate the overlap between CRF, TH- and pro-DYN-immunopositive cells and OXA containing fibers. Figure 7 shows close opposition of OXA-immunoreactive fibers and CRF-, TH and pro-DYN-immunoreactive pericarya. Distributed throughout the NTS-A_2_ catecholaminergic cell group, and intermingled with TH-containing cells numerous OXA-immunoreactive axons were found (Figure 7A). In shell region of the NAc OXA containing varicosities were observed in close apposition with pro-DYN-containing neurons (Figure 7C). At more caudal level, in the BNST, thin OXA-containing fibers have been revealed in overlap with CRF-containig neurons (Figure 7B). OXA-immunoreactive axons juxtaposed to CRF-containing cells were present in the CeA (Figure 7E). OXA-immunoreactive axons densely innervated the hypothalamic paraventricular nucleus and were found in in close apposition to CRF neurons in the medial parvocellular subdivision. (Figure 7D).

### OX1R antagonism decreases CeA CRF activity during naloxone-precipitated morphine withdrawal

**Figure 8:**
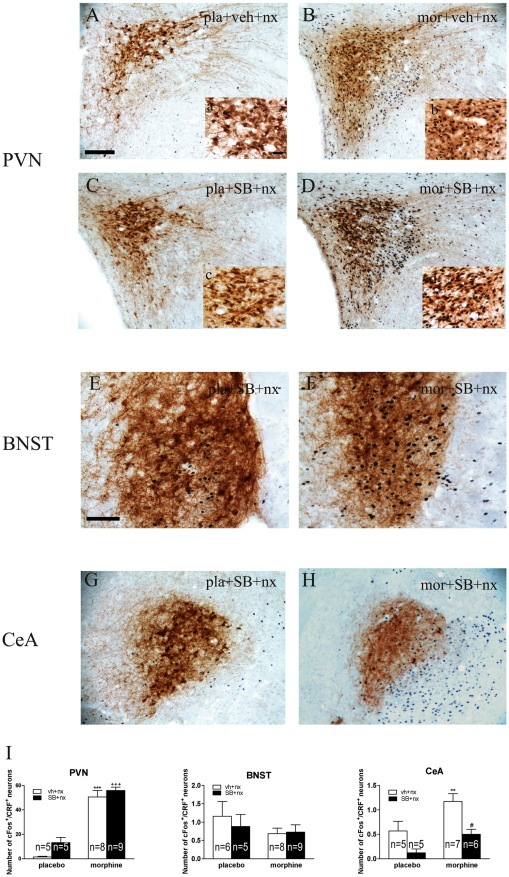
OX1R antagonist SB334867 attenuates activation of CRF-containing neurons in the CeA but not in either the PVN or the BNST. Microscopy showing double-label immunohistochemistry for c-Fos and CRF in the PVN (A–D), BNST (E, F) and CeA (G, H). (a–d): higher magnification from (A–D). Scale bars, 100 µm (A–H); 50 µm (a–d). (I): mean ± SEM. **p<0.01, ***p<0.001 versus placebo + vehicle + naloxone; ^+++^p<0.001 versus placebo + SB334867 + naloxone; ^#^p<0.05 versus morphine + vehicle + naloxone.

We also tested the influence of the OX1R antagonist SB334867 on activation of CRF neurons (as assessed by double immunostaining with anti-c-Fos and anti-CRF antibodies) in the PVN, BNST, and CeA (Figure 8). Two-way ANOVA for c-Fos expression in CRF neurons in the PVN revealed a main effect of morphine treatment [F(1,23) = 123.63; p<0.0001] but no significant effect either of SB334867 pretreatment or “pretreatment” X “SB treatment” interaction. The two-way ANOVA for c-Fos expression in CRF neurons in the BNST showed no significant effect of morphine pretreatment, SB334867 administration or “pretreatment” X “SB treatment” interaction. The two-way ANOVA for c-Fos expression in CeA CRF neurons revealed main effect of morphine pretreatment [F(1,21) = 9.96; p = 0.0048], SB334867 administration [F(1,21) = 12.92; p = 0.0017] but no interaction between morphine pretreatment and SB334867 treatment. *Post hoc* analysis revealed that morphine withdrawal increased (p<0.001) the number of CRF-containing neurons expressing c-Fos in the PVN (n = 8), which indicates an activation of CRF neurons, compared with placebo controls receiving naloxone (n = 5). In these neurons, there was no effect of SB334867 administration on c-Fos expression (Figure 8A–D, I; n = 9). In the CeA (Figure 8G, H, I), there was a significant increase in c-Fos expression in CRF-positive neurons (p*<*0.01; n = 7). The SB334867 pretreatment significantly attenuated (p<0.05; n = 6) the increase in c-Fos expression in CRF CeA neurons, which indicates that there is a greater OXA input to CeA CRF neurons during morphine withdrawal.

### OX1R blockade does not antagonize naloxone-precipitated morphine withdrawal-induced HPA axis activation

**Figure 9:**
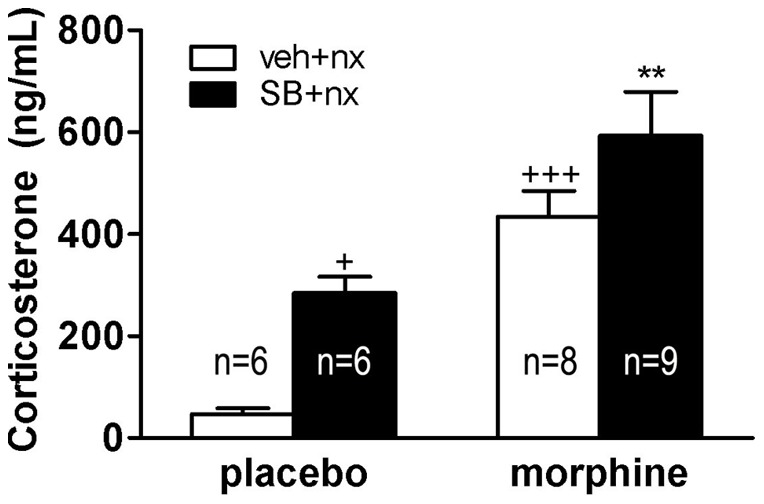
Effects of OX1R antagonism on the morphine withdrawal-induced increase in plasma corticosterone levels. SB334867 did not attenuate the plasma corticosterone response to naloxone-induced morphine withdrawal. Data represent the mean ± SEM of corticosterone concentration 120 min after naloxone (nx) injection to placebo- or morphine-treated rats receiving vehicle (veh) or SB334867 (SB) 20 min before naloxone administration. Mean ± SEM. ^+^p<0.05, ^+++^p<0.001 versus placebo + vehicle + naloxone **p<0.01 versus morphine + vehicle + naloxone.

We measured plasma corticosterone concentrations (as HPA axis activation marker) in blood samples obtained from morphine-dependent or control rats 2 h after injection of naloxone. Two-way ANOVA for corticosterone revealed significant effect of chronic morphine treatment [F(1,25) = 29.72; p<0.0001] and significant effect of acute treatment [F(1,25) = 9.64; p = 0.0047]. As shown in Figure 9, plasma corticosterone levels increased significantly (p<0.001) in vehicle pretreated, morphine withdrawn rats. To evaluate if there is a link between OX1R activation and HPA axis hyperactivity during morphine withdrawal, plasma corticosterone concentrations were measured in animals made dependent on morphine and pretreated with SB334867 before naloxone administration. Corticosterone levels in SB334867 plus naloxone-treated morphine-pelleted animals were significantly (p<0.01; n = 9) higher than those observed in the placebo group also administered SB334867 plus saline (n = 6). No significant differences were seen between morphine-dependent rats receiving vehicle before naloxone and those receiving SB334867. In addition, placebo-pelleted rats receiving SB334867 showed significant (p<0.05) higher plasma corticosterone concentrations compared with its control receiving vehicle instead of SB334867 (n = 6).

## Discussion

This report provides evidence in rats of the critical involvement of orexinergic system via OX1R in activation of brain stress system during morphine withdrawal.

In agreement with previous data [14], [15], present results show an important role for OXA and OX1R pathways in the somatic expression of opiate withdrawal as well as in molecular changes within the brain stress systems induced by morphine withdrawal. Naloxone administration to morphine dependent rats induced robust withdrawal symptoms. Our results indicate that the blockade of OX1R significantly decreased several of the somatic signs of naloxone-precipitated morphine withdrawal as well as the global score, suggesting that activation of OX1R pathways might positively modulate the somatic expression of opiate withdrawal. These data are in agreement with previous results showing that administration of SB334867 directly into LC to rats attenuates signs of morphine withdrawal [16]. In addition, mice lacking the pre-propeptide encoding for orexins or pretreated with SB334867 display markedly reduced symptoms of opiate withdrawal [14], [15]. In the present study we demonstrate that in rats, the involvement of OXA in morphine withdrawal somatic symptoms is mediated, at least in part, by OX1R.

Previous study on orexin-T-LacZ reporter mice indicated that morphine withdrawal stimulates the transgene expression. Using quantitative real time PCR measurement, here we provide a direct evidence for activation of OXA gene expression in the rat hypothalamus during morphine withdrawal. Furthermore, OXA mRNA levels peak between 30–60 min after naloxone administration. This timing of transcriptional activation is compatible with CRE-mediated transcription as it has been shown in the orexinergic neurons of morphine-withdrawn CRE-LacZ reporter mice [14]. Indeed, naloxone-precipitated morphine withdrawal results in activation of orexin neurons in the lateral hypothalamus as revealed by immunocytochemical detection of c-Fos protein. In contrast to the situation seen in morphine withdrawn mice, cell in all the three functionally distinct subpopulation of hypothalamic orexinergic neurons became c-Fos positive upon morphine withdrawal in rats.

Heterogeneity in orexin neurons has been described in previous animal studies [17]: orexinergic neurons located in the DMH and PFA have been suggested to control arousal and modulate stress responses, whereas neurons in the LLH project to areas related to positive reinforcing effects of drugs. We have confirmed activation of orexin neurons in the DMH and PFA [14], [15], however, for the first time, demonstrate that morphine withdrawal induces c-Fos expression in orexinergic neurons in the LLH subdivision. This result suggests that LLH may also be a critical region contributing to morphine withdrawal response in rats. The disparities between our recent data and previous studies might originate from species difference (rat vs mouse) or from methodological differences, since escalating morphine doses in mice were used by Sharf et al. (2008) [15].

Interestingly, present work also shows hyperactivation of the brain stress system during morphine withdrawal that was attenuated by SB334867. Given that the PVN receives orexinergic inputs arising from both the PFA and the LLH subdivisions of the LH [18], our data might suggest the involvement of OXA neurons arising not only from the DMH and PFA, but also from the LLH, in the activation of the brain stress system during opiate withdrawal.

Both the HPA axis and the extended amygdala are dysregulated by chronic administration of drugs of abuse [10], [19], [20]. Furthermore, acute drug withdrawal may also increase the activity of noradrenergic pathways innervating the CeA and the PVN [20]–[22]. All these processes are involved, at least in part, in the negative motivational states during drug withdrawal. Presents results show an enhancement of c-Fos expression during morphine withdrawal in the main nuclei of the extended amygdala, and in the PVN, the apex of the HPA axis. The NTS-A_2_ cell group, the main noradrenergic cell group innervating the brain stress system [8], is also activated after naloxone-precipitated opiate withdrawal. Present data shows that SB334867 administration attenuates this activation of the extended amygdala and the PVN, suggesting that the orexinergic system, via OX1R, might play a main role in regulating the activation of the brain stress systems during acute opiate withdrawal. The presence of orexinergic fibers as well as OX1R and/or OX2R within all these nuclei showed in the present work and others [4] supports this hypothesis [23]. The present study shows that the OXA system provides anatomical input to the brain stress system, including the extended amygdala, the PVN and the NTS. Light microscopic double immunocytochemical analysis revealed close apposition between OXA fibers and CRF-, pro-DYN- and TH-expressing in the PVN, CeA, NAc and NTS respectively. These findings provide an anatomical basis for potential modulation of CRF, noradrenergic and DYN neurons by OXA.

NAc shell has been related to the expression of morphine withdrawal somatic symptoms [15], together with the CeA and BNST [24], [25]. Supporting this hypothesis, present results showed that attenuation of somatic signs during opiate withdrawal was accompanied by a decrease of c-Fos expression in these sites.

All major drugs of abuse stimulate the HPA axis during acute withdrawal [10], [19]. The PVN receives OXA and OXB projections, both of them originating in the PFA and LLH, although OXA immunoreactive fibers are found in higher densities than those of OXB. Regarding OX receptors, OX2R mRNA is predominant in the PVN [18]. However, conflicting findings have been published concerning the activity of the orexinergic system on the HPA axis [18], [26]. The results of the present study show that morphine withdrawal induced an increase in corticosterone release, which was not blocked by SB334867 administration. When we examined the action of SB334867 on the activity of the CRF neurons in the PVN during morphine withdrawal, we found that both the morphine-dependent rats injected with SB334867 or with vehicle showed similar responses to naloxone injection. Since there was an attenuation of c-Fos expression in the PVN after SB334867 administration in morphine-withdrawn rats, and our results show that this decrease does not occur in the CRF neurons, it could be suggested that the activity of other PVN neurons, such as AVP-containing cells (which also participates in ACTH and then in glucocorticoids release), is inhibited by the OX1R antagonist. Given that OX2R mRNA is predominantly expressed in the PVN [23], and that the administration of OX2R antagonist to rats inhibits ACTH release induced by OXA or stress [18], it might be postulated that the action of OXA at this level would be mediated mainly by the OX2R. Thus, it seems logical that the responsiveness of the PVN CRF neurons and the HPA axis to morphine withdrawal did not change after blocking OX1R. However, SB334867 administration induced a slight activation of CRF neurons in the PVN and glucocorticoids release in control rats. Since OXA binds to OX1R and OX2R with similar affinity [1], and we have blocked OX1R, OXA might bind exclusively to OX2R. Thus, OX2R stimulation in the PVN would result in CRF-containing cells activation and in glucocorticoids release. Furthermore, it is well established that orexins exert an influence on the regulation of the hypothalamus-pituitary-adrenal axis, acting both on its central peripheral branch [27].

Numerous studies have supported the importance of the brainstem noradrenergic afferents in regulating the brain stress system during opiate withdrawal [8], [22], [25], [28]. Since SB334867 administration did not block the activation of the NTS, our data do not support a role of the OX1R pathways in regulating the activity of the noradrenergic A_2_ cell group.

Extended amygdala nuclei and their neurotransmitter systems have been related with the positive reinforcing effects of drugs, as well as with the negative reinforcing effects of drugs withdrawal [10]. It has been previously reported that OXA has an excitatory action on the CeA [29], and that this activation occurs in CRF-containing neurons [30]. Accordingly, our results show an increase in c-Fos expression in CRF-positive neurons in the CeA during naloxone-induced morphine withdrawal, which was blocked by SB334867. These data indicate the involvement of the orexinergic system in modulating the response to opiate withdrawal syndrome in the CeA via OX1R.

The BNST neurons are innervated by orexin-immunoreactive fibers originating in the LH [4], [5], and display moderate levels of OX1R expression. [23]. In addition, BNST receive noradrenergic inputs originating in the NTS [25]. Present data show that BNST CRF neurons were not activated after naloxone administration to morphine dependent rats, which is in agreement with previous data showing no increase in c-Fos expression in BNST CRF neurons in morphine-withdrawn rats [31]. Just like in the PVN, SB334867 administration did not alter the response of BNST CRF neurons to morphine withdrawal. Should be taken into account is that these two nuclei receive important noradrenergic inputs from the NTS [28], [32], whose activation in morphine-withdrawn rats is not attenuated by the OX1R antagonist.

In summary, this study provides evidences of activation of orexinergic in the three subdivisions of the LH during naloxone-induced morphine withdrawal that is accompanied with increased OXA mRNA transcription and increased OXA input to brain stress system. Furthermore, present data reveal a critical involvement of the orexinergic system in the physical symptoms of opiate abstinence syndrome, supporting a therapeutic potential of OX1R antagonists in addictive disorders, as has been proposed [15], [33]. Present findings highlight the pivotal role of orexins in the activation of the hypothalamic and extrahypothalamic brain stress systems during opiate withdrawal in rats.

## Methods

### Animals

Male Wistar rats (220–240 g, at the beginning of the experiment; Harlan, Barcelona, Spain) were housed 2–3 per cage, in a room with controlled temperature (22±2°C) and humidity (50±10%), with free access to water and food (Harlan Teklad standard rodent chow; Harlan Interfauna Ibérica, Barcelona, Spain). Animals were adapted to standard 12-h light-dark cycle (lights on: 08:00–20:00 h) for 7 days before the beginning of the experiments. For the behaviour study rats were housed individually in the same room. Allsurgical and experimental procedures were performed in accordance with the European Communities Council Directive of 24 November 1986 (86/609/EEC), and approved by the local Committees for animal research (REGA ES300305440012, Murcia and Institutional Animal Care and Use Committee of the Institute of Experimental Medicine, Budapest). The study was approved by the University of Murcia bioethics committee (RD 1201/2005) and Ministerio de Ciencia y Tecnología (SAF2009-07178), Spain.

### Drug treatment and experimental procedure

Rats were made dependent on morphine by subcutaneous (sc) implantation of two 75 mg slow-release morphine pellets (provided by the Ministerio de Sanidad, Madrid, España) for 6 days under light ether anaesthesia. Control rats received placebo pellets containing the excipient without morphine. This procedure has been shown to produce constant plasma morphine concentration starting a few hours after implantation of the pellets and morphine dependence as revealed by expression of full withdrawal syndrome after acute injection of opioid antagonists [34]. Dependence on morphine (as measured by withdrawal response) is achieved 24 h after implantation of pellets and remained constant for 15 days [35]. Six days after the implantation of morphine or placebo pellets, precipitated withdrawal was induced by s.c. injection of naloxone (1 mg/kg; in a volume of 1 mL/kg body weight).

The experimental conditions investigated for the different assays were: orexin gene expression: (i) placebo + saline (n = 6), (ii) placebo + naloxone (n = 5), (iii) morphine + saline (n = 6), (iv) morphine + naloxone 15 min (n = 4), (v) morphine + naloxone 30 min (n = 4), (vi) morphine + naloxone 60 min (n = 5), (vii) morphine + naloxone 120 min (n = 4); activation of the orexinergic neurons of the LH and the number of orexinergic neurons during morphine dependence and withdrawal: (i) placebo + saline 120 min (n = 5), (ii) placebo + naloxone 120 min (n = 5), (iii) morphine + saline 120 min (n = 5), (iv) morphine + naloxone 120 min (n = 7); opiate withdrawal-induced physical signs of dependence, c-Fos expression in the brain stress system, activation of the CRF neurons of the BNST, PVN and CeA and corticosterone release: (i) placebo + vehicle + naloxone 120 min (n = 6), (ii) placebo + SB334867 + naloxone 120 min (n = 5), (iii) morphine + vehicle + naloxone 120 min (n = 8), (iv) morphine + SB334867 + naloxone 120 min (n = 9).

### Measurement of the withdrawal syndrome

Experiments were carried out in a quiet room. The observer was unaware of the drug combination used. Rats were individually placed into transparent plastic cages 15 min before the naloxone injection and observed continuously for the occurrence of somatic signs of opiate withdrawal up to 30 min after the naloxone injection, at which time most of the acute behavioral effects manifest [36]. Subsequently, previously identified behavioral characteristics of the rat opiate withdrawal [37] were evaluated including: wet-dog shakes, jumping, paw tremor, teeth chattering, mastication, ptosis, piloerection, sniffing, writhing, tremor and diarrhoea. The number of wet-dog shakes, jumping, sniffing, and paw tremor was counted as the number of events occurring during the total test time period (graded signs). Teeth chattering, body tremor, mastication, ptosis, piloerection and diarrhoea were scored 1 for appearance or 0 for non-appearance within each 5 min time. To obtain a comprehensive index of the severity of somatic opioid withdrawal including all the signs examined, a global withdrawal score was calculated for each animal by giving each individual sign a relative weight as previously reported [38]: jumping x 0,8; wet dog shakes x 1; paw tremor x 0,35; sniffing x 0,5; writhing x 0,5; ptosis x 1,5; teeth chattering x 1,5; body tremor x 1,5; diarrhoea x 1,5; mastication x 1,5 and piloerection x 1,5.

Body weight loss was determined as the difference between the weight determined immediately before naloxone injection and a second determination made 2 h later. The weight gain of the rats was checked during treatment to ensure that the morphine was liberated correctly from the pellets because it is known that chronic morphine treatment induces a decrease in body weight gain due to lower caloric intake [20], [39].

In order to investigate the effect of the OX1R blockade on the physical symptoms of morphine withdrawal, rats were pretreated with the selective OX1R antagonist SB334867 [40], at the dose of 20 mg/kg i.p., 20 min prior saline or naloxone injection. This dose of SB334867 is consistent with other recent studies [15], [41]. Vehicle was delivered at the same volume as the SB334867 solution. The somatic signs of opiate withdrawal were evaluated up to 30 min after naloxone injection. The following parameters were determined two hours after saline or naloxone administration: c-Fos immunoreactivity, plasma corticosterone and ACTH levels.

### Primer design

Primers used for the comparative C_T_ (threshold cycle) experiments were designed by the Primer Express 3.0 program. Primer sequences were the following:

*Orexin-A:*

Forward: TCCTTCAGGCCAACGGTAAC

Reverse: GGCAGGGATATGGCTCTAGCT

*GAPDH:*

Forward: ACAGCCGCATCTTCTTGTGC

Reverse: GCCTCACCCCATTTGATGTT

### Dissection of whole hypothalamic samples

After decapitation at different time-points (15, 30, 60 and 120 min), the brain was rapidly removed from the skull and placed to an RNase free rubber surface and the cerebellum was removed. The boundaries of the hypothalamic blocks were at the optic chiasm in rostral- at the mammillary bodies in caudal- and at the hypothalamic sulcus in the lateral directions. The samples were immediately frozen on dry ice and stored at −70°C until assay.

### Quantitative real-time PCR

Total RNA was isolated from hypothalamic samples with QIAGEN RNeasy MiniKit (Qiagen, Valencia, CA, USA) according the manufacturer's instruction. To eliminate genomic DNA contamination DNase I treatment was used (100 ml Rnase free DNase I (1 uDNase I, Fermentas) solution was added). Sample quality control and the quantitative analysis were carried out by NanoDrop (Thermo Scientific). Amplification was not detected in the RT-minus controls. cDNA synthesis was performed with the High Capacity cDNA Reverse Transcription Kit (Applied Biosystems, Foster City, CA, USA). The designed primers (Invitrogen) were used in the real-time PCR reaction with Power SYBR Green PCR master mix (Applied Biosystems, Foster City, CA, USA) on ABI StepOne instrument. The gene expression was analyzed by ABI StepOne2.0 program. The amplicon was tested by Melt Curve Analysis on ABI StepOne instrument. Experiments were normalized to GAPDH expression.

### Perfusion and Immunohistochemical detection of OXA and c-Fos

Two hours after naloxone or saline injections, rats were deeply anesthetized with pentobarbital (100 mg/kg ip) and quickly perfused through the ascending aorta with saline followed by ice-cold fixative (paraformaldehyde 4%). Brains were post-fixed in the fixative for 3 h and then placed in PBS containing 30% sucrose overnight. Series of 30 μm frontal sections were cut on freezing microtome, collected in cryoprotectant and stored at −20° C until processing. After blocking with H_2_O_2_ and normal goat serum (Sigma, St Louis, MO, USA) for c-Fos or normal rabbit serum for OXA immunostaining, tissue sections were incubated in the following primary antibodies: rabbit anti-c-Fos (1∶10000, Santa Cruz Biotechnology, Santa Cruz, CA, USA) or goat anti-orexin-A (1∶2000, Santa Cruz). This was followed by application of a biotinylated anti-rabbit or anti-goat IgG (Vector Laboratories, Burlingame, CA, USA), and then with the avidin–biotin complex. Visualization of the antigen–antibody reaction sites was performed using 3, 3′-diaminobenzidine (DAB, Sigma) nickel intensification for c-Fos or DAB chromogen only for OXA. Sections were mounted onto chrome-alumn gelatine coated slides, dehydrated through graded alcohols, cleared in xylene and cover slipped with dibutylphtalate (DPX).

### Double-labelling immunohistochemistry of c-Fos-immunoreactive nuclei and OXA-positive neurons or CRF-positive neurons

For double-labelling, tissue sections from each rat in each treatment group were processed for c-Fos immunoreactivity using DAB nickel intensification and then OXA or CRF were revealed using DAB chromogen only. Briefly, c-Fos immunostaining was performed as described above. Following the c-Fos staining, sections were rinsed in PBS, treated with bovine albumin serum or goat serum and then incubated with the rabbit polyclonal anti-OXA antibody or the rabbit policlonal anti-CRF antibody (1∶1000, a generous gift from Wylie Vale, The Salk Institute, La Jolla, CA, USA). The same immunohistochemistry procedures described above were followed. The OXA- or CRF-antibody-peroxidase complex was developed in DAB. The sections were mounted onto chrome-alumn gelatine coated slides and coverslipped.

### Quantification of c-Fos immunoreactivity

Images were captured by means of DM4000B Leica microscope (Leica, Madrid, Spain) equipped with a video camera (DFC290; Leica). The distribution of c-Fos-positive cell nuclei was plotted using a computer assisted image analysis system (QWIN; Leica, Madrid, Spain). The boundaries of the NTS, the BNST, the CeA, the shell of the NAc and the PVN were outlined and the number of immune-positive profiles was recorded after thresholding the images to a common level. The number of c-Fos nuclear profiles within the confines of cell groups of interest was counted bilaterally in complete series of sections and estimates were corrected for double-counting errors. To avoid observer bias, all sections were quantified by a blinded investigator. Total counts for different brain regions are expressed as mean ± SEM.

### Quantification of OXA-positive cells and c-Fos/OXA or c-Fos/CRF double stained profiles

Images were captured by means of DM4000B Leica microscope (Leica) equipped with a video camera (DFC290; Leica). The LH was further subdivided into its three sub-populations: DMH, PFA and LLH of the LH, and we measured the number of OXA neurons in the three subpopulations. After labelling the area of interest by a computer assisted image analysis system (QWIN; Leica, Madrid, Spain) using identical rectangular frame (195 μm side) first, single labelled OXA neurons were manually counted (X 20 magnification) in LH. Then, c-Fos/OXA and double stained neurons were identified as profiles with blue/dark nuclear staining in addition to brown cytoplasmic deposits. Four to six sections from each rat were counted bilaterally at distinct rostro-caudal levels of the LH and averaged to obtain a single value for each rat. c-Fos-positive CRF (PVN, BNST, and CeA) double-stained neurons were counted in the same way. All the counting was made by a single researcher, who was blinded to all treatment information.

### Double-labelling immunohistochemistry of OXA fibers and DYN- TH- or CRF-positive neurons

For double-labelling, tissue sections from each placebo-treated rats were processed for OXA fibers immunoreactivity using DAB nickel intensification and then pro-dynorphin (DYN), CRF or TH were revealed using DAB chromogen only. Briefly, OXA fibers immunostaining was performed as described above. Following the OXA fibers staining, sections were rinsed in PBS, treated with 3% bovine serum albumin and then incubated with the guinea-pig anti-DYN antibody (1∶2000, Neuromics, Edina, MN, USA), rabbit anti-CRF antibody or anti-TH antibody (1∶6000, Chemicon, Temecula, CA). The same immunohistochemistry procedures described above were followed. The DYN- and CRF- and TH-antibody–peroxidase complex was developed in DAB. The sections were mounted onto chrome-alumn gelatine coated slides and coverslipped.

### Radioimmunoassay

Two hours after saline or naloxone injection, rats were decapitated (between 1100 and 1200 h to avoid circadian variations in plasma levels of the hormones). Trunk blood was collected, plasma was separated and levels of corticosterone were measured by commercially available kits for rats (125I-corticosterone RIA; MP Biomedicals, Orangeburg, NY). The sensitivity of the assay was 7.7 ng/mL.

## Materials

Pellets of morphine (75 mg morphine base/pellet; Alcaliber Labs., Madrid, Spain) or lactose (placebo) were prepared by the Department of Pharmacy and Pharmaceutics Technology (School of Pharmacy, Granada, Spain); naloxone HCl was purchased from Sigma, dissolved in sterile 0.9% NaCl (saline) and administered in volumes of 0.1 ml/100 g body weight. DMSO was purchased from Sigma. SB334867 (1-(2-methylbenzoxazol-6-yl)-3-[1], [5]naphthyridin-4-yl urea hydrochloride; Tocris, Bristol, UK) was suspended in 2% DMSO and 10% 2-hydroxypropyl-β-cyclodextrin (Sigma, St. Louis, MO) in sterile water; and was given in a volume of 5 mL/kg (i.p.). Drugs were prepared fresh every day.

### Statistical analysis

Data are presented as mean ± S.E.M. Data were analysed using two-way or one-way (when required) analysis of variance (ANOVA) followed by a *post hoc* Newman–Keuls test. Differences with a P-value <0.05 were considered significant.
